# bioGWAS: A Simple and Flexible Tool for Simulating GWAS Datasets

**DOI:** 10.3390/biology13010010

**Published:** 2023-12-23

**Authors:** Anton I. Changalidis, Dmitry A. Alexeev, Yulia A. Nasykhova, Andrey S. Glotov, Yury A. Barbitoff

**Affiliations:** 1 Department of Genomic Medicine, D.O. Ott Research Institute of Obstetrics, Gynaecology, and Reproductology, 199034 St. Petersburg, Russia; anton@bioinf.me (A.I.C.); yulnasa@gmail.com (Y.A.N.); 2 Bioinformatics Institute, 197342 St. Petersburg, Russia; dm_aleksee@mail.ru; 3 Faculty of Software Engineering and Computer Systems, ITMO University, 197101 St. Petersburg, Russia

**Keywords:** GWAS, SNP, simulation, pathway analysis

## Abstract

**Simple Summary:**

Genome-wide association studies (GWAS) are a powerful tool for the identification of genes affecting human traits. Still, the interpretation of GWAS results is complicated, and new tools are actively being developed. Due to the scarcity of available datasets, simulation of GWAS data with known genetic effects is important as it enables accurate evaluation of such tools. In this study, we developed a flexible tool, bioGWAS, that provides a set of important functionalities for simulating GWAS results. We demonstrate that bioGWAS can efficiently generate GWAS results with predefined causal genes and biological processes and is capable of recapitulating the results of published GWAS studies. We thus believe that bioGWAS is an excellent method for testing bioinformatics software for GWAS results processing, as well as for the generation of datasets for educational purposes.

**Abstract:**

Genome-wide association studies (GWAS) have proven to be a powerful tool for the identification of genetic susceptibility loci affecting human complex traits. In addition to pinpointing individual genes involved in a particular trait, GWAS results can be used to discover relevant biological processes for these traits. The development of new tools for extracting such information from GWAS results requires large-scale datasets with known biological ground truth. Simulation of GWAS results is a powerful method that may provide such datasets and facilitate the development of new methods. In this work, we developed bioGWAS, a simple and flexible pipeline for the simulation of genotypes, phenotypes, and GWAS summary statistics. Unlike existing methods, bioGWAS can be used to generate GWAS results for simulated quantitative and binary traits with a predefined set of causal genetic variants and/or molecular pathways. We demonstrate that the proposed method can recapitulate complete GWAS datasets using a set of reported genome-wide associations. We also used our method to benchmark several tools for gene set enrichment analysis for GWAS data. Taken together, our results suggest that bioGWAS provides an important set of functionalities that would aid the development of new methods for downstream processing of GWAS results.

## 1. Introduction

Understanding how genetics influence the traits of an individual (phenotype) is a crucial task of modern biology. This task is particularly challenging for traits that are affected by multiple genetic and environmental factors, also known as complex traits. Studying the role each gene plays in the development of complex traits can provide insights into the relevance of various biological processes for these traits. This knowledge has far-reaching implications, such as helping in the early detection and prevention of severe diseases [1]. One of the methods that may help to identify the genes and processes involved in a particular trait is the Genome-Wide Association Study (GWAS).

GWAS is used to investigate the relationship between changes in the DNA sequence (genetic variants) and complex traits [2]. A typical GWAS study involves genotyping and phenotyping of a large cohort of individuals (for binary traits, this cohort is split into case and control subgroups) followed by a statistical analysis of the association between the presence of a particular genetic variant and the trait. Such a statistical analysis yields a set of so-called summary statistics that include *p*-values and effect size estimates for each of the studied genetic variants.

In addition to the identification of genetic variants that demonstrate statistically significant association with the phenotype, GWAS data can be used to identify pathways and processes involved in the studied trait. Gene set and pathway enrichment analyses serve as invaluable tools for this task. A rich set of tools has been developed to perform such an analysis using GWAS results (e.g., DEPICT [3], MAGMA [4], or Pascal [5]). Application of these methods allows for the statistical association of specific traits with particular biological processes within an organism, thereby offering a nuanced understanding of genotype-phenotype interactions [6].

Even though multiple tools for pathway analysis in GWAS data have been developed, unbiased benchmarking of these tools remains challenging due to the lack of datasets with a known set of ground truth pathways involved in a trait. An optimal dataset for this task should be relatively large and include extensive genetic and phenotypic information, along with summary statistics of genome-wide association analysis. Availability of all of these data types is essential for testing the accuracy, power, and other statistical properties of the developed instruments for downstream processing of GWAS results, including pathway analysis methods [7].

There are several sources of large-scale genotype and phenotype information that are commonly used in the development and testing of new approaches to GWAS result annotation. These include 1000 Genomes Project (1KGP) [8] and the UK Biobank (UKB) [9,10], both of which have collected and analyzed large amounts of genetic data. 1KGP data are freely available; however, these do not include any phenotypic information (apart from the ethnicity and geographical origin of the participants). UKB provides rich phenotype information; however, access to the UKB data requires submitting an application and payment of an access fee. More importantly, none of the widely used data sources can provide any ground truth knowledge about molecular pathways involved in complex traits, making their usage for the benchmarking of enrichment analysis (EA) methods complicated. Yet another problem with any real dataset that contains human genotypes and phenotypes is the inability to easily share it due to privacy concerns. Beyond research, this limitation may also negatively affect education in the field of GWAS.

There is another option to obtain GWAS datasets—namely, to simulate them. Popular methods for such simulations are HAPGEN2 [11], TriadSim [12], and simGWAS [13]. HAPGEN2 samples from existing haplotypes; however, it is limited to generating genotype data for case-control studies. TriadSim also takes into account realistic linkage disequilibrium (LD) structure; however, it requires existing GWAS data and is specifically designed to work with parent-offspring trios. simGWAS simulates GWAS summary data directly, which increases the speed of computation. However, it is also impossible to control all the parameters of the gene-traits association, and input of real genotype data is complicated.

Here, we propose a novel, simple, and flexible pipeline that can simulate GWAS data with different patterns of associations (with specific variants, random variants, and variants from specific gene sets/pathways), parameters of heritability, and effect size distribution. The developed instrument also includes preprocessing and postprocessing steps, along with visualization of the results. We believe that our tool, bioGWAS, will be useful for the generation of complex, biologically meaningful GWAS datasets for both research and educational purposes.

## 2. Materials and Methods

### 2.1. Overview of bioGWAS

In this work, we introduce a novel algorithm for simulating comprehensive GWAS data, which has been both implemented and extensively tested. Our algorithm generates realistic data that includes genotypes, phenotypes, and the summary statistics of their associations. Furthermore, an automatic visualization component was developed that enables users to effortlessly assess the accuracy and appropriateness of the generated data.

**Figure 1:**
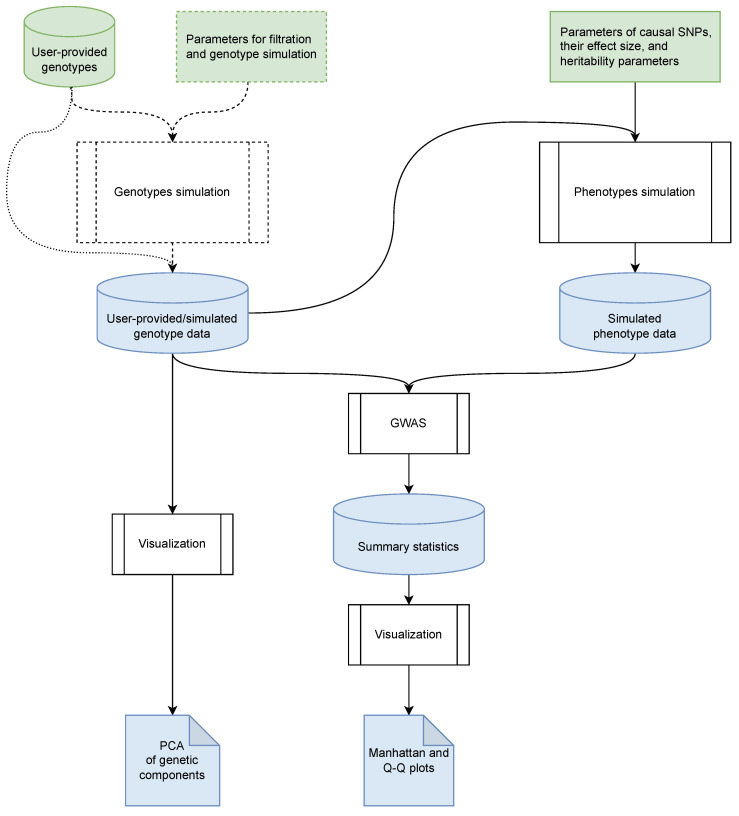
The basic steps of the bioGWAS workflow. Here, the green color is used to mark inputs (cylinders for data, rectangles for parameters), the white rectangles are used to denote algorithm steps, and the blue color is used to mark outputs (cylinders for data, rectangles with curved corners for plots). The dotted line indicates alternative steps.

Our tool, bioGWAS, employs an algorithm composed of three steps (see Figure 1): generating genotyping data, generating the corresponding phenotypic data (traits), and performing analysis of associations between the generated genotypes and phenotypes. The detailed workflow, including the file formats utilized and the programs employed, is illustrated in Supplementary Figure S1. Each step of the workflow will be described in subsequent sections.

### 2.2. Genotype Simulation

The first step of the bioGWAS algorithm is generating genotype data. This step can be omitted if the user prefers to use available genotype data directly for phenotype simulation and GWAS. If the user opts to generate the genotypes, a reference panel of genotypes has to be provided (1KGP phase3 genotypes are recommended as the default option) along with the desired sample size (*N*). To ensure data quality, a data cleaning procedure is included. The procedure involves:

1. Filtering variants based on MAF, thus excluding rare variants (this step is independent of further filtering of causal variants based on both minimum and maximum MAF values);
2. Filtering samples based on a sample identifier list, which allows users to specify a list of samples for analysis, streamlining the process and eliminating the need for manual filtering.

Genotype filtering is performed using maf and keep options of PLINK2 v2.00a4.9LM [14].

In order to achieve an expanded sample size, the algorithm utilizes HAPGEN2 v2.2.0 [11] to simulate the desired number of haplotypes from a reference panel (default value is one thousand individuals). The simulation process includes random permutations to generate these genotypes from the available input data. Given the substantial sizes of files containing genotype data (ranging from several Gb to several Tb), data for each chromosome is recommended to be processed separately during this step (to achieve this, per-chromosome input genotypes are expected). Subsequently, upon completion of the simulation, the data files are merged using PLINK2 pmerge-list function.

### 2.3. Phenotype Simulation

Next, the bioGWAS proceeds to simulate the phenotypic data. Phenotype simulation relies on the predefined set of causal variants or pathways specified by the user. If the latter option (drawing causal variants from a pathway) is used, causal variants from the selected gene sets are drawn randomly based on the generated (or user-provided) genotypes. Additionally, random variants are added (based on the desired proportion of causal variants from the gene set of interest). The exact steps of the algorithm are as follows:

1. The closest function of the BEDTools v2.30.0 tool [15] is used to annotate each SNP according to the genome annotation file provided by the user. This step collects information about the closest gene for each variant. Additionally, variants are filtered by minimum and maximum MAF. By default, all common variants ( 0.05< MAF <0.5 ) are considered as potentially causal (i.e., minimum MAF =0.05 and maximum MAF =0.5 ), though the user may specify custom thresholds to specify the desired MAF window for causal variants.
2. Next, the user-defined number of causal variants (*K*) are selected. Depending on the user input, this is done either based on the user-specified gene set (pathway) or an explicitly provided set of causal variants.
  In the former case, variants are drawn from the gene-SNP mapping obtained in the first step. Let there be *n* genes in the specified gene set, and *k* variants from this set are set to be causal out of a total of *K* causal variants affecting the trait ( k≤K ). Then, K−k causal variants should be drawn from random genes.
  The simulation algorithm then looks as follows:
  (a) *k* genes are randomly selected from the *n* genes in the gene set of interest. Gene set information is taken from the file in a standard gene matrix transpose (GMT) format, in which each row contains a gene set name accompanied by a comprehensive list of corresponding genes (for human data, curated gene sets from the Molecular Signatures Database (MSigDB) [16] is used by default);
  (b) Based on the gene-SNP mapping obtained in step (1), *k* variants corresponding to the genes from step (2a) are selected (one SNP per gene; if the number of genes in the set is less than the value of *k*, then more than one SNP per gene may be selected, maintaining uniform coverage);
  (c)
K−k random variants (not belonging to the *n* genes from causal gene sets) are added.
  Thus, this procedure yields *K* causal variants to be used in phenotype simulation, *k* of which correspond to the given set of genes.
  In the case of manually specified causal variants, the final set of causal variants is constructed by filtering out user-provided variant IDs using the list of variants present in the genotype data.
3. Individual genotypes for the constructed set of causal variants from step (2) are extracted from the genotype data file.
4. Finally, phenotype data are simulated using genotypes at the causal variant loci obtained in step (3). Continuous trait values are generated using phenotypeSimulator (R-package, v.0.3.4) [17]. The following simulation parameters can be specified: the mean effect size ( $m_{\beta }$ ), the standard deviation of effect size ( $sd_{\beta }$ ), the genetic effects variance (heritability, $h^{2}$ ), the proportion of the genetic variants’ effect variance ( $h_{s}^{2}$ ), the proportion of variance of shared genetic variant effects ( θ ), the proportion of genetic variant effects to have a trait-independent fixed effect ( $p_{indep}$ ), the proportion of observational noise effect variance ( ϕ ), and the variance of shared observational noise effect ( α ). The description of these parameters can be found in the PhenotypeSimulator documentation (https://cran.r-project.org/web/packages/PhenotypeSimulator/PhenotypeSimulator.pdf, accessed on 23 October 2023).

### 2.4. Association Analysis

The final step of the workflow involves the statistical testing of the associations between each variant in the generated samples and the corresponding phenotype values. The association analysis is performed using PLINK1.9 assoc function [18], yielding a summary statistics file, which contains detailed information on each variation along with their respective association statistics (i.e., effect size estimates and association *p*-values).

### 2.5. Visualization of Generated GWAS Results

There are different types of visualization that are routinely used in the GWA studies. In bioGWAS, several common plots are automatically generated, including (i) scatterplots of individual genotypes in the space of principal components obtained through principal component analysis (PCA), and (ii) quantile-quantile (Q-Q) plots and Manhattan plots of the association *p*-values obtained on the final stage of the pipeline.

In order to assess the distribution of both initial and simulated genotypes and the patterns of population stratification, PCA is performed on both simulated and initial genotype data, with additional LD pruning using the indep-pairwise function in PLINK2. The first four components of the PCA are plotted on scatterplots, enabling examination of the sample clustering patterns.

Generation of Q-Q and Manhattan plots in bioGWAS is based on the summary statistics derived from the association analysis. These plots can be used to evaluate the strength of the associated signal. Manhattan plots help highlight the significantly associated loci and variants. For the creation of Manhattan and Q-Q plots, bioGWAS employs the CMplot v.4.2.0 [19] R package. Additionally, PLINK1.9’s clump function is used to highlight loci, rather than individual variants, on the Manhattan plot. This function groups significant loci based on patterns of LD and the distance between variants (default clumping parameters are used).

### 2.6. Validation of Simulation Results

In order to ensure high accuracy of data simulation, we made an effort to fit the default parameters of phenotype simulation. For this purpose, a series of experiments were conducted for parameter selection. 1KGP [8] genotype dataset was used for experiments (genome assembly version: Genome Reference Consortium Human Build 37 (GRCh37)). A list of sample identifiers and their corresponding populations of origin was also acquired from the project website and used to select 503 samples representing individuals of European ancestry. Furthermore, the MAF>0.05 filter was used during the simulation process to exclude rare variants.

Then, the simulation of phenotypes was conducted using the generated genotypes. Causal variants were selected based on gene annotations and information about the associations of genes with biochemical processes. Comprehensive gene annotations were obtained from the GENCODE website for GRCh37 in Gene Transfer format (GTF) for GENCODE release 19 [20]. Information on pathway affiliations was obtained from the MSigDB [16]. 186 pathway gene sets derived from the Kyoto Encyclopedia of Genes and Genomes (KEGG) database were used [21,22,23].

The following parameters were explored for phenotypic simulation:

- Number of causal variants: K∈{10,20,30} ;
- Number of causal variants from a given set of genes: $k=\frac{K}{2}$ ;
- For each *K*, 120 combinations of parameters of PhenotypeSimpulator were evaluated (see Section 2.3 and Supplementary Table S1).

After running bioGWAS for each combination of parameters, the GWAS summary statistics were processed using PLINK1.9 clump function to merge variants into loci based on LD patterns and distance between variants. Then, we evaluated the proportion of SNP clumps containing the predefined causal variants as well as the proportion of clumps containing these causal variants. The former value corresponds to the precision of the analysis, while the latter corresponds to recall (sensitivity). These values were then used to calculate the F1-score: $F_{1}=\frac{2}{recall^{-1}+precision^{-1}}$ . Since the F1-score represents both metrics (calculated as the harmonic mean of precision and recall), it was chosen as the main metric for comparison and selection of the best-performing parameter sets.

In the first round of validation, one dataset was simulated for each parameter set (full simulation results are available in Supplementary Table S1). For the second round, we selected several best-performing parameter sets. At least two sets were selected for each *K*; in addition, average $F_{1}$ scores across all tested values of *K* were calculated to identify universally efficient settings to be used as default ones. In this round, 20 simulations were run for each parameter combination. The mean value of each metric was calculated across the 20 replicates, and the best parameter set was selected as the default. Further experiments were conducted using this set. It should be noted, however, that the parameter evaluation was performed only for *N* = 10,000. Further adjustments of simulation parameters may be needed with larger or smaller *N*.

### 2.7. Replication of Existing GWAS Datasets

Pre-computed summary statistics of UKB round 2 (http://www.nealelab.is/uk-biobank, accessed on 25 February 2022) and FinnGen (FG) [24] release 6 (https://r6.finngen.fi/, accessed on 25 February 2022) data were used for an additional in silico experiment by simulation of GWAS data with a similar set of significantly associated loci. Traits with code M06 (other rheumatoid arthritis) and I9_HYPTENSPREG (hypertension complicating pregnancy, childbirth, and the puerperium) were used from UKB and FinnGen data, respectively. 1KGP dataset processed similarly to Section 2.6 was used for simulation. Index variants at each locus were selected as causal ones for data simulation. We then compared original and simulated summary statistics, significant loci, and plots generated by bioGWAS.

### 2.8. Using bioGWAS to Benchmark Pathway Analysis Tools

The final step was the assessment of the utility of bioGWAS for benchmarking pathway analysis tools for GWAS. To conduct such an analysis, GWAS datasets with a known causal relationship to biological pathways with varying numbers of member genes were simulated. Three such pathways were chosen: (i) small (KEGG_STEROID_BIOSYNTHESIS), which includes 17 genes; (ii) medium (KEGG_PPAR_SIGNALING_PATHWAY), which includes 69 genes; and (iii) big (KEGG_FOCAL_ADHESION), which includes 199 genes. For each of these three cases, 30 simulations were performed, each incorporating the selection of 30 causal variants, with 15 of these SNPS belonging to the pathway of interest. In addition, 30 simulated GWAS datasets with fully random causal variants were created to estimate the type I error rate.

Enrichment analysis using these simulated datasets was performed using the widely-used MAGMA [4] and Pascal [5] tools. Three different models inside MAGMA were used (principal components regression (linreg), the SNP-wise mean (snp-wise=mean), and SNP-wise top -1 (snp-wise=top)), and default settings were applied for Pascal.

After obtaining EA results, we calculated the true positive rate (TPR) for each of the pathways, defined as the percentage of simulations in which the target pathway was identified as significantly enriched by the corresponding method. For simulations involving fully random causal variants, we calculated the false positive rate (FPR) as the percentage of simulations where at least one pathway emerged as significant.

### 2.9. bioGWAS Implementation and Data Availability

The bioGWAS pipeline is written in the Snakemake pipelining language and is freely available for users at https://github.com/toharhymes/bioGWAS/ (accessed on 11 December 2023). Both plain and containerized versions of the tool are available. Additional data and code used for the analysis presented in further sections are available in the same repository.

## 3. Results and Discussion

### 3.1. Validation of Data Simulated Using bioGWAS

As described in the previous section, our proposed method, bioGWAS, generates simulated genotypes, simulated phenotypes, GWAS summary statistics, and plots that can be used to explore the results of the simulation (Figure 1). After the initial implementation of the method, we set off to validate the results of the simulation using the aforementioned output files.

First, we assessed the results of genotype simulation in bioGWAS. Supplementary Figure S2 shows the scatter plots of the genotypes (for real 1KGP individuals and 1000 simulated individuals derived from 1KGP data) in space of the first four principal components. The results of this analysis showed that, while a certain degree of population stratification can be observed in the original genotyping data, the sample of simulated individuals is much more homogeneous. This observation is expected, given the nature of genotype simulation by random haplotype permutation. Thus, simulated genotypes are better suited for further association analysis, even though such a homogeneous structure of the sample makes simulated data less similar to a real population.

Having validated the results of genotype simulation, we next went on to evaluate the performance of further pipeline stages, including simulation of phenotype data and association analysis. In the pursuit of selecting the most appropriate simulation parameters, an evaluation of the 360 parameter sets was performed using a two-step procedure, and the ability of bioGWAS to simulate data with a predefined set of causal variants was evaluated for each parameter set (Supplementary Tables S1 and S2). This analysis showed that, indeed, bioGWAS demonstrates high levels of both precision and recall with proper parameter selection. The accuracy of simulation results mostly depended on the ratio of mean effect size to its standard deviation ( $m_{\beta }$ / $sd_{\beta }$ ) and on the average proportion of trait variance tagged by each of the selected causal variants ( $h^{2}$ /*K*). When the aforementioned values were optimal (e.g., $m_{\beta }$ / $sd_{\beta }>10$ and $h^{2}$ / K≈0.01 ), the results of the analysis proved the ability of bioGWAS to generate GWAS results with high values of precision and recall of causal SNP identification (Supplementary Figures S3 and S4). With a lower number of causal variants ( K=10 ), an average $F_{1}>0.95$ was achieved in several cases (in particular, for the following parameter set chosen as the default one: $m_{\beta }=0.05$ , $sd_{\beta }=0.001$ , $h^{2}=0.1$ , $h_{s}^{2}=1.0$ , θ=0.0 , $p_{indep}=1.0$ , ϕ=1.0 , α=0.0 ). With more causal variants ( K≥20 ), the performance metrics were slightly lower, but an average recall of 0.99 could be achieved under certain settings (see Supplementary Table S2).

**Figure 2:**
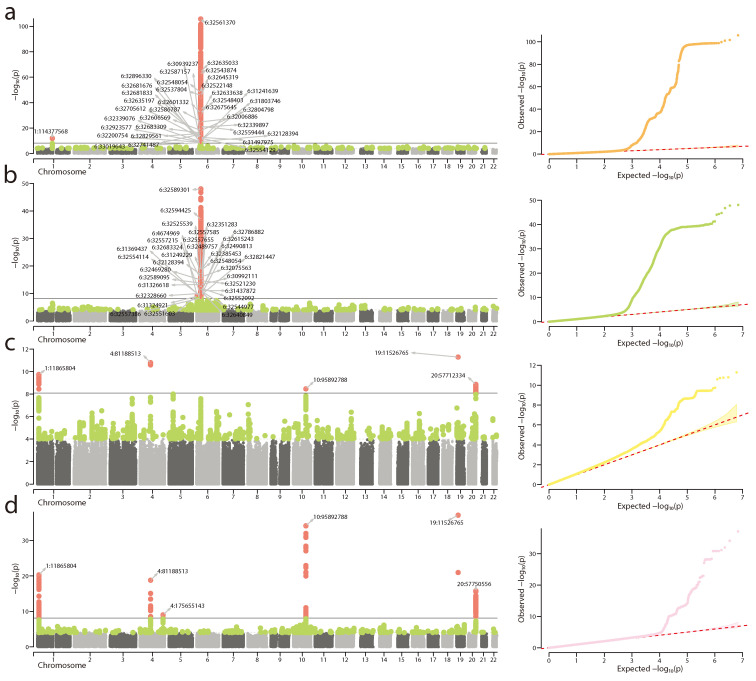
Recapitulation of real GWAS datasets with bioGWAS. Shown are Manhattan (on the left) and Q-Q (on the right) plots for (**a**) original UKB GWAS for “other rheumatoid arthritis”; (**b**) simulated GWAS based on causal variants from the UKB GWAS for “other rheumatoid arthritis”; (**c**) original FG GWAS for “hypertension complicating pregnancy, childbirth, and the puerperium”; (**d**) simulated GWAS based on causal variants from the FG GWAS for “hypertension complicating pregnancy, childbirth, and the puerperium”. On the Manhattan plots, green color indicates variants with $p<10^{-4}$ , red color indicates genome-wide significant associations.

To further validate bioGWAS we tested its ability to recapitulate the results of real GWAS using only a set of the corresponding index variants at genome-wide significant loci. To do so, we simulated GWAS datasets based on the sets of index variants obtained from summary statistics of GWAS for “other rheumatoid arthritis” and “hypertension complicating pregnancy, childbirth, and the puerperium” from the UKB and FG datasets, respectively. Manhattan and Q-Q plots for original and simulated data are presented in Figure 2. Although the Q-Q plots look the same and indicate the presence of strong genome-wide association signals for both original and simulated data, the sets of significant loci slightly differ between the original data and simulation results.

For other rheumatoid arthritis (Figure 2a), multiple associations within the HLA region are observed in the real data, and these associations remained significant in simulation results (Figure 2b). However, a single association outside of MHC (on chromosome 1) has not shown up in the simulation results (perhaps, due to a much higher p-value in the original dataset). Although this result may seem disappointing, it is well aligned with the fact that not all true causal variants become significant during the association studies and shows that simulated data can be as noisy as the real one. In the case of the second trait used in evaluation (where the original p-values for index variants were much more similar), all loci that were significant in the original dataset also passed the significance threshold in simulation results, and the simulated dataset contained an additional significant locus.

Taken together, the results of the validation suggest that bioGWAS can well simulate homogeneous genotype datasets and efficiently partition the simulated trait heritability between user-defined causal genetic variants. Hence, we conclude that the datasets generated by bioGWAS can be used for the evaluation of bioinformatic methods of GWAS results processing.

### 3.2. Using bioGWAS to Benchmark Pathway Analysis Tools for GWAS Data

Finally, having demonstrated the ability of bioGWAS to simulate realistic GWAS datasets with the expected genetic architecture, we went on to use the results provided by bioGWAS to evaluate the performance of commonly used tools for pathway analysis with GWAS data—MAGMA [4] and Pascal [5]. To this end, we simulated a total of 90 sets of summary statistics with three different biological pathways from KEGG selected as the source of causal genetic variants (see Section 2 for the details of the procedure). In addition, another 30 sets of summary statistics were generated using fully random causal variants.

**Figure 3:**
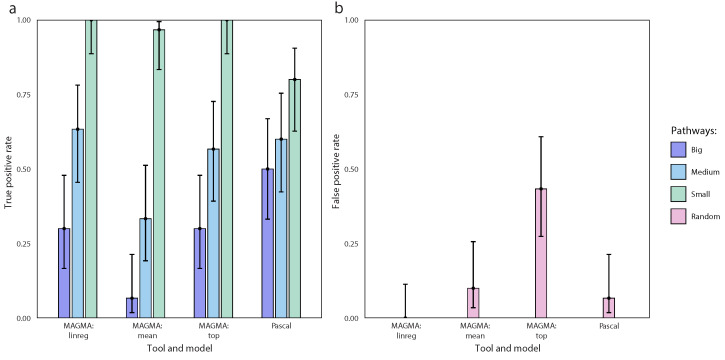
Results of benchmarking of the commonly used EA tools for GWAS data, MAGMA and Pascal, using the datasets simulated with bioGWAS. (**a**) true positive rates (power) for identifying the enrichment of KEGG pathways (small—KEGG_STEROID_BIOSYNTHESIS (17 genes), medium—KEGG_PPAR_SIGNALING_PATHWAY (69 genes), big—KEGG_FOCAL_ADHESION (199 genes)); (**b**) false positive rates (the probability of obtaining sifnificant results with random causal variants). Error bars correspond to the Wilson score interval.

For each tool, we calculated its power to detect the enrichment and the rate of type I errors (true positive and false positive rates). TPR was assessed as the proportion of simulations in which the specified causal pathway was identified by the tool. FPR was estimated as the rate of statistically significant pathways identified in a dataset with random causal variants. The results are presented in Figure 3. It can be seen that for all models, a reasonably high TPR is achieved only when almost all genes from a gene set bear causal genetic variants (15 causal genes out of 17 were selected in KEGG_STEROID_BIOSYNTHESIS, termed “small” pathway). As the proportion of causal genes to the total number of genes in the pathway decreases, TPR decreased significantly for all models. Although all methods showed a non-zero TPR for all pathways tested, MAGMA’s mean method performed much worse compared to Pascal and other alternative models in MAGMA. At the same time, Pascal performed significantly better for large gene sets while maintaining a relatively low FPR. Of the four tested models, only MAGMA’s top model demonstrated high FPR (Figure 3b).

Although a more systematic benchmarking of pathway analysis tools for GWAS is indeed required to make a final conclusion regarding the accuracy of existing tools, we believe that the results presented above further prove the utility of bioGWAS for the developers of methods for downstream processing of GWAS results.

## 4. Conclusions

Taken together, we introduce a novel tool, bioGWAS, specifically designed to simulate biologically meaningful GWAS datasets. bioGWAS can help during the development of new methods for GWAS results processing. Furthermore, bioGWAS may be helpful for simulating datasets that can be used for educational purposes. bioGWAS offers a large number of settings as well as output files, which evidence its advantages over competing methods (Table 1). Not only does the developed tool offer precise simulation capabilities, but it has also been fine-tuned with default parameters carefully optimized to capture the desired biological features of simulated traits. Thus, we believe that bioGWAS has great potential for its application in future genetic studies, offering other researchers a powerful tool for the simulation of GWAS data.

## Figures and Tables

**Table 1:** Comparison of simulation algorithms for GWAS data.

Algorithm	HAPGEN2 [11]	TriadSim [12]	simGWAS [13]	bioGWAS
Supplied as	Command line interface (CLI)	R package	R package	CLI with a Docker image
Input format	Known haplotypes in .haps/.legend files	Genotypes in a PLINK binary file, must include trios	Genotype matrix ^†^	Genotypes in a phased VCF or a PLINK binary file
Genotype filtration	Manual (as a preprocessing step)	Manual (as a preprocessing step)	Manual (in R code, or in pre-processing)	Automatic, for samples and variants (cutoffs specified as parameters)
Setting causal variants.	Yes	Yes	Yes	Yes
Setting gene sets and biological pathways	Partially (as a list of manually selected variants)	Partially (as a list of manually selected variants)	Partially (as a list of manually selected variants)	Yes: as a list of variants, or as names of biological pathways/gene sets
Outputs	Genotype data, summary statistics for causal variants	Genotypes and quantitative trait samples	Summary statistics	Genotype data, phenotype data; summary statistics; visualizations (genotypes PCA, Q-Q, and Manhattan plot)

Table cells colored in green correspond to tools with best (most comprehensive/flexible) functionality for each parameter. ^†^—-a number of R code manipulations need to be made to read data from VCF or other formats.

## Data Availability

All data and code pertinent to the results presented in this work are available at https://github.com/TohaRhymes/bioGWAS/.

## Supplementary Materials

The following supporting information can be downloaded at: www.mdpi.com/xxx/s1, Figure S1: A detailed schematic representation of the bioGWAS workflow; Figure S2: Scatterplots of the original (first row) and simulated (second row) set of genotypes over the first four principal components; Figure S3: Relationships and dependencies between Recall, Precision, F1 score, and phenotype simulation parameters; Figure S4: Dependence of Precision and Recall in simulated GWAS datasets with different phenotype simulation parameters; Table S1: Parameters and results of the first round of validation simulation; Table S2: Parameters and aggregated results of the second round of validation simulation.

## Abbreviations

The following abbreviations are used in this manuscript:

GWAS	Genome-wide association study
SNP	Single-nucleotide polymorphism
EA	enrichment analysis
UKB	UK Biobank
FG	FinnGen
1KGP	1000 Genomes Project
MAF	Minor Allele Frequency
GRCh37	Genome Reference Consortium Human Build 37
