# Supplementary material for: bioGWAS: A Simple and Flexible Tool for Simulating GWAS Datasets
Source: Biology (Basel). 2023 Dec 23;13(1):0. doi: 10.3390/biology13010010 (PMC11154295; doi:10.3390/biology13010010)
Supplement: Supplementary file 1 [file biology-13-00010-s001.zip › Supplementary_Figures.pdf]

Supplementary figures

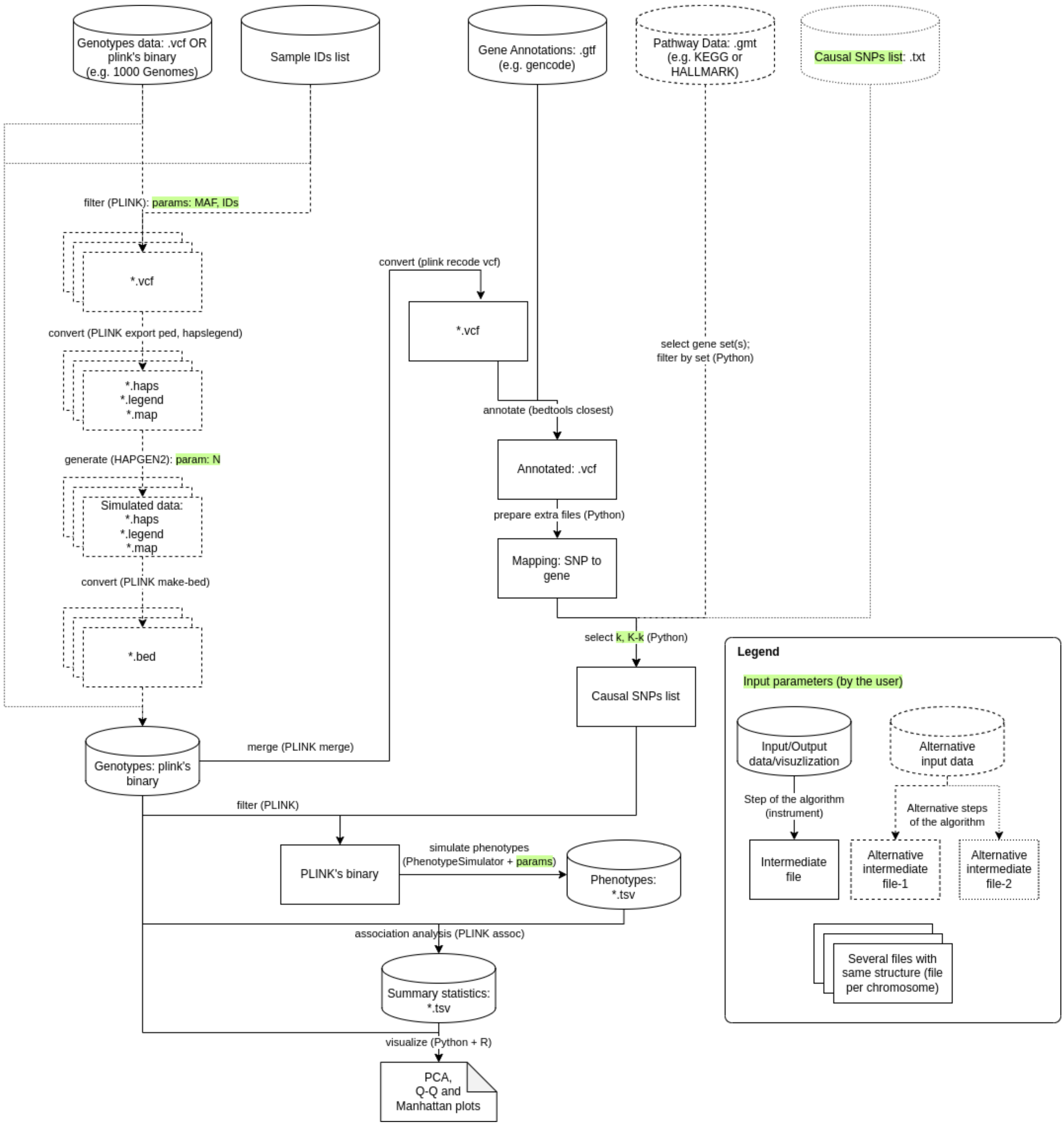

Supplementary Figure S1 – A detailed schematic representation of the bioGWAS workflow. The cloud is used to represent the data obtained from internet sources. The green elements represent user-defined parameters. The stacked rectangles represent parallel processing of file groups, such as partitioning by chromosomes.

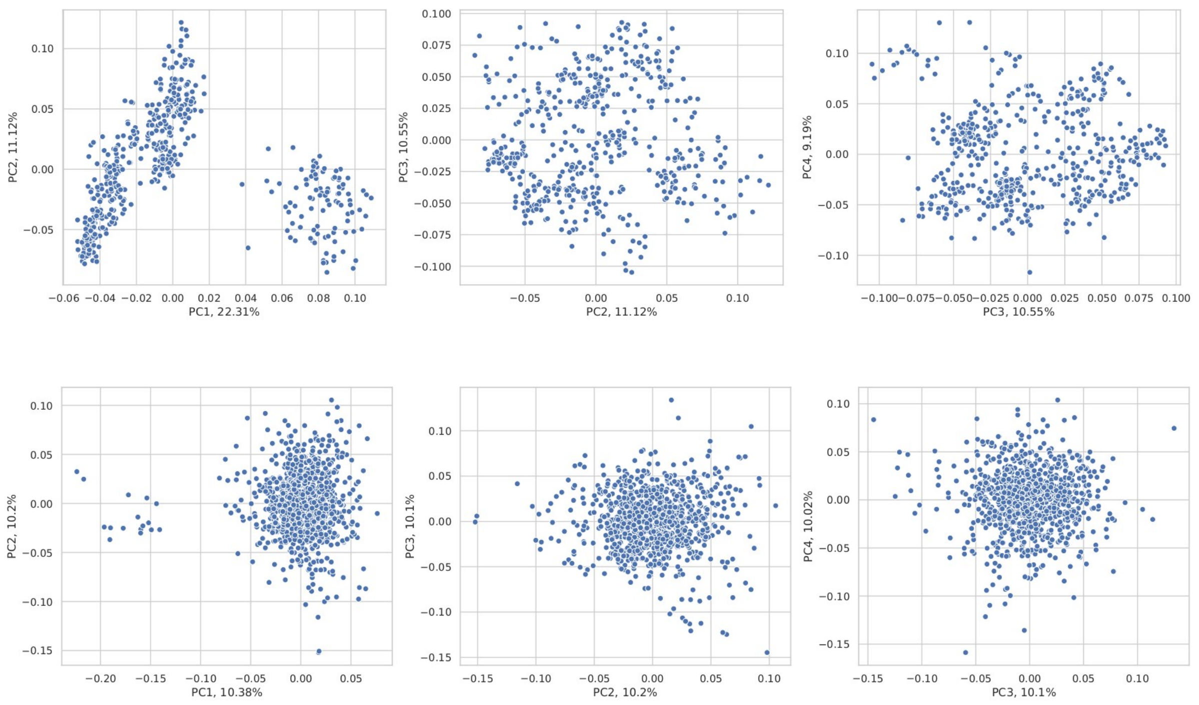

Supplementary Figure S2 – Scatterplots of the original (first row) and simulated (second row) set of genotypes over the first four principal components. Simulated data are more homogeneous. The percentage of explained variance (calculated with respect to the first ten PCs) is also indicated next to the component number on the axes.

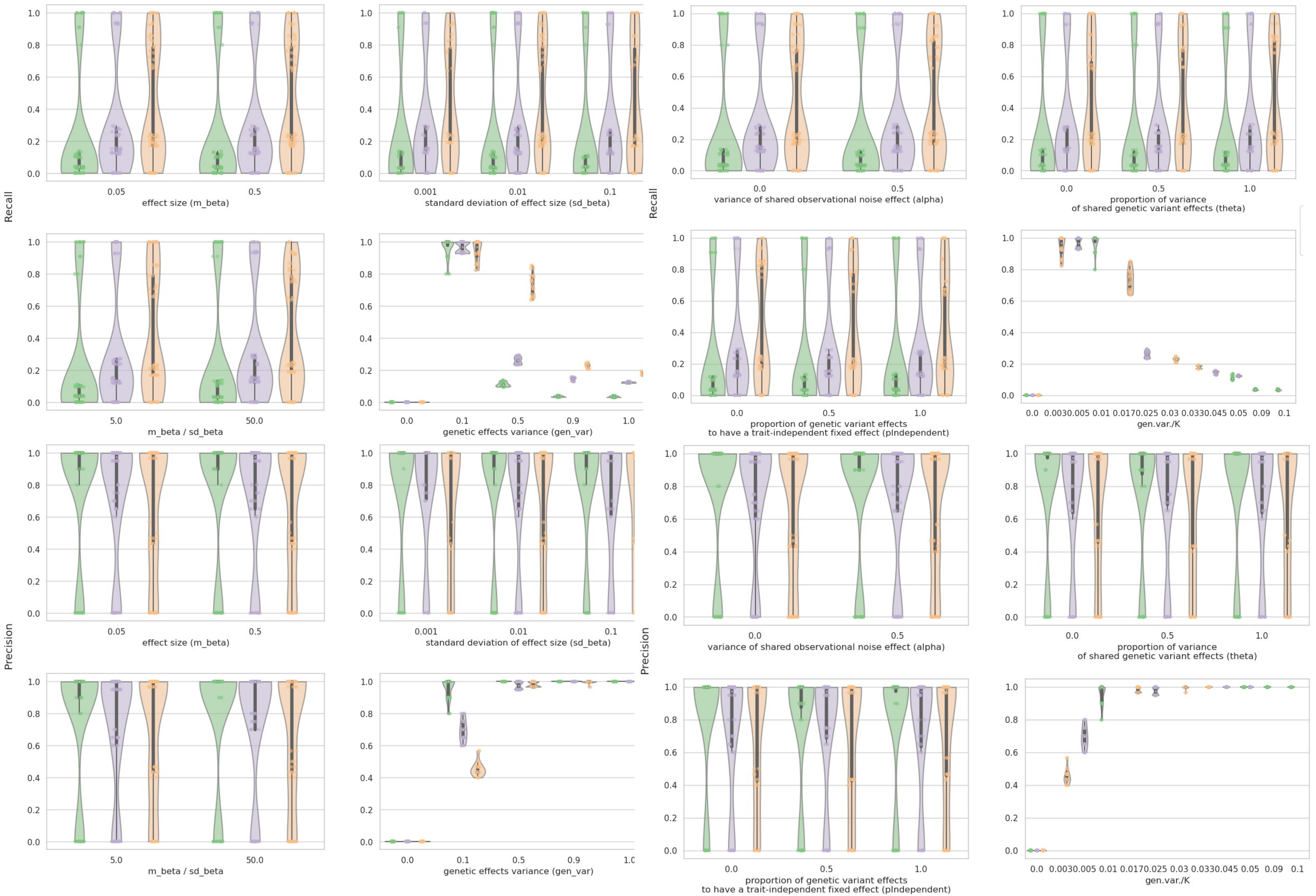

Recall
Precision
1.0
0.8
0.6
0.4
0.2
0.0
0.05
0.5
effect size (m_beta)
0.001
0.01
0.1
standard deviation of effect size (sd_beta)
0.0
0.5
variance of shared observational noise effect (alpha)
0.0
0.5
1.0
proportion of variance
of shared genetic variant effects (theta)
5.0
50.0
m_beta / sd_beta
0.0
0.1
0.5
0.9
1.0
genetic effects variance (gen_var)
0.0
0.5
1.0
proportion of genetic variant effects
to have a trait-independent fixed effect (pIndependent)
0.0 0.0030.005 0.01 0.0170.025 0.03 0.0330.045 0.05 0.09 0.1
gen.var./K

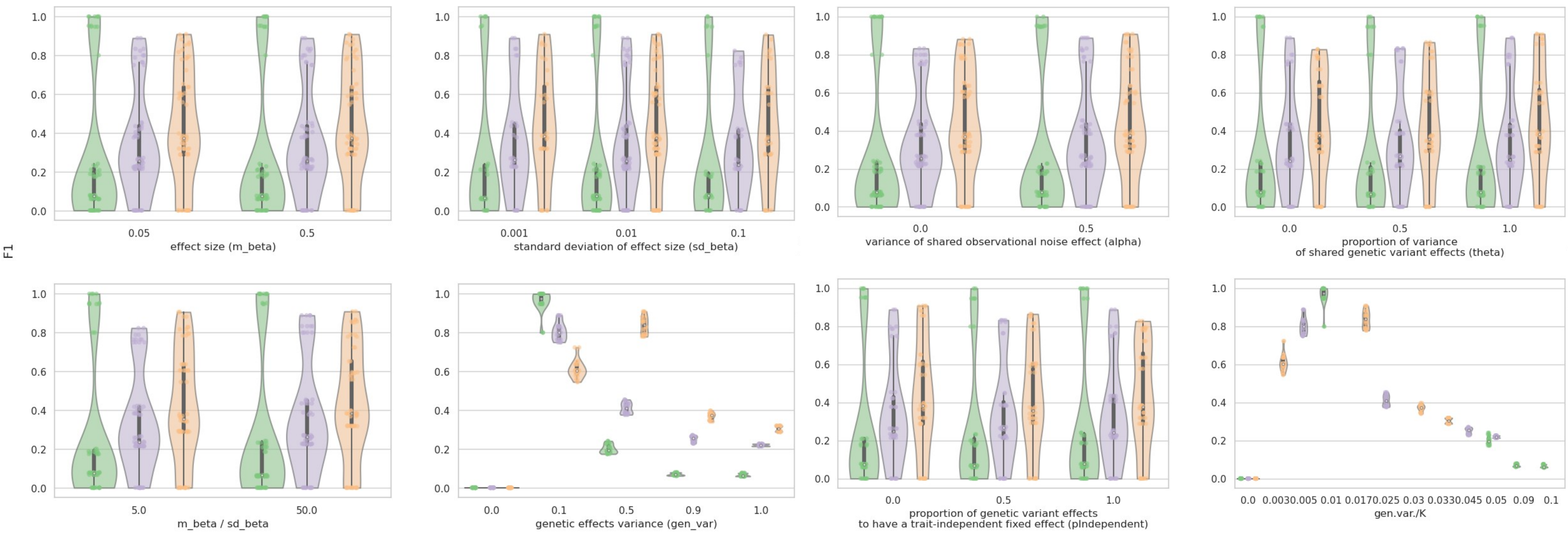

Supplementary Figure S3 – Relationships and dependencies between Recall (first 2 rows), Precision (third and fourth row), F1 score (last two rows), and phenotype simulation parameters: effect size (m_beta), standard deviation of effect size (sd_beta), their ratio (m_beta/sd_beta), genetic effects variance (gen_var), variance of shared observational noise effect (alpha), proportion of variance\nof shared genetic variant effects (theta), proportion of genetic variant effects\nto have a trait-independent fixed effect (pIndependent), gen.var.-to-K ratio, amount of causal SNPs (K); k=K/2.

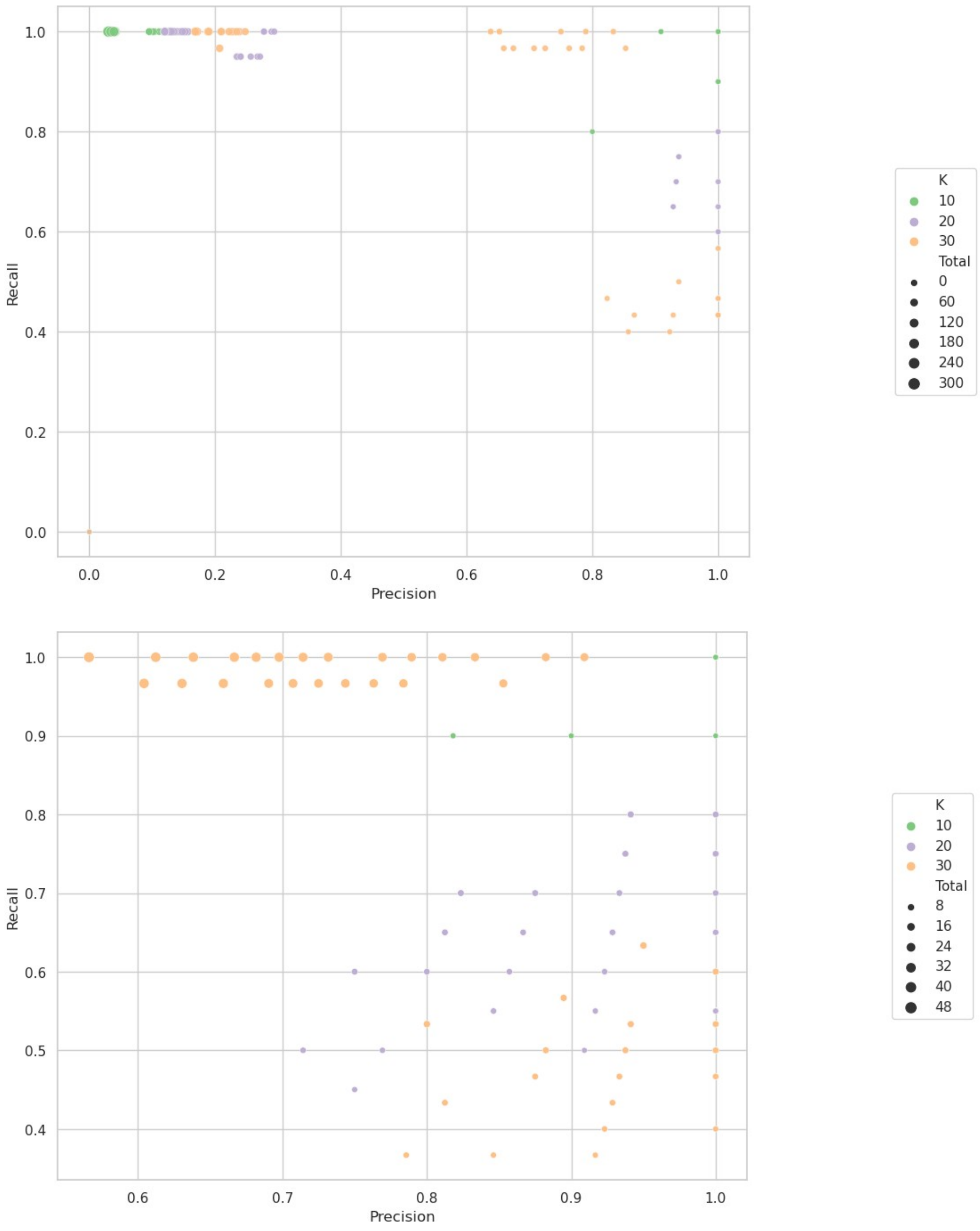

Supplementary Figure S4 – Dependence of Precision and Recall in simulated GWAS datasets with different phenotype simulation parameters. Results for a) first round of best parameters selection (trying out 120 parameter set per each *K*); and b) second round of the best parameters selection. The best case – both of these characteristics are equal to 1 (And therefore $F_1=1$).
